# The prospective relation between eating behaviors and BMI from middle childhood to adolescence: A 5-wave community study

**DOI:** 10.1016/j.pmedr.2022.101795

**Published:** 2022-04-19

**Authors:** Oda Bjørklund, Lars Wichstrøm, Clare Llewellyn, Silje Steinsbekk

**Affiliations:** a Department of Psychology, Norwegian University of Science and Technology (NTNU), Dragvoll 7491 Trondheim, Norway; b St Olav University Hospital, 7030 Trondheim, Norway; c Department of Behavioural Science & Health, University College London, 1-19 Torrington Place, London, WC1E 7HB, UK

**Keywords:** ALT-SR, Autoregressive latent trajectory model with structured residuals, BMI, Body Mass Index, CEBQ, Children’s Eating Behaviour Questionnaire, FIML, Full Information Maximum Likelihood, SDQ, Strengths and Difficulties Questionnaire, TESS, Trondheim Early Secure Study, Eating behaviors, Obesogenic eating, BMI, Child, Adolescent, Longitudinal

## Abstract

Some eating behaviors are associated with increased risk of childhood obesity and are thus potential targets for obesity prevention. However, longitudinal research, especially on older children and adolescents, is needed to substantiate such a claim. Using data from a representative birth cohort of Norwegian children followed up biennially from age 6 to age 14 (analysis sample: n = 802), we tested if change in eating behaviors predicts increased body mass index (BMI) throughout childhood and adolescence, or if it is the other way around; higher BMI predicting more obesogenic eating. Eating behaviors were measured using the Children’s Eating Behaviour Questionnaire (CEBQ) and BMI was measured objectively using digital scales. To separate within-person- and between-person effects and control for all time-invariant confounders (i.e., variables that do not change over the study period), we applied an autoregressive latent trajectory model with structured residuals (ALT-SR). Results showed that increases in obesogenic eating behaviors did not predict higher BMI at any age. It was the other way around: Increased BMI predicted increases in food responsiveness and emotional overeating at all time points, and enjoyment of food from 8 to 10 years and from 10 to 12 years. Furthermore, increased BMI predicted decreases in satiety responsiveness at all time points except from age 12 to age 14, as well as diminished emotional undereating from 12 to 14 years. One implication of our findings, if replicated, is that targeting obesogenic eating behaviors to change weight outcomes may be less effective in children older than age 6.

## Highlights

• Does change in eating behaviors predict prospective change in BMI, or vice versa?
• More obesogenic eating behaviors did not predict higher BMI from age 6 to age 14.
• Rather, higher BMI predicted higher levels of obesogenic eating behaviors over time.
• Targeting eating behaviors to prevent obesity may be less effective after age 6.

## 1 Introduction

Childhood obesity is of high public concern and associated with numerous health risks (Han et al., 2010). The way children behave towards food, affecting how much, what and when they eat, is considered important in understanding the etiology of childhood obesity (Carnell & Wardle, 2008) and is captured by individual differences in eating behaviors (French et al., 2012). Eating behaviors are related to self-regulation of energy intake, and some eating behaviors cause people to overeat (McCrickerd, 2018). Therefore, eating behaviors have been considered important to address in prevention and treatment of pediatric obesity (Carnell and Wardle, 2008, Kral et al., 2018). Such interventions typically include efforts to help parents apply feeding practices that promote infants’ self-regulation of eating (Daniels et al., 2009, Daniels et al., 2013, Daniels et al., 2015, Harris et al., 2020), and improve children’s ability to recognize internal signals of hunger and fullness and adjust their food intake accordingly (Boutelle et al., 2020, Boutelle et al., 2014, Johnson, 2000).

To be effective targets of interventions, eating behaviors must be involved in the etiology of obesity. Even though etiology cannot be proven by observational designs, a prerequisite for having an etiological role is that alleged obesogenic eating behaviors do predict increased weight status in children. More specifically, children who eat in response to external cues such as the sight and smell of food (i.e., high food responsiveness), are interested in and enjoy meals (i.e., high enjoyment of food), eat more and not less in response to negative emotions (i.e., high emotional overeating, low emotional undereating), are less sensitive to internal signals of fullness (i.e., low satiety responsiveness), and have a higher eating speed (i.e., low slowness in eating) (Wardle et al., 2001) are expected to increase their weight more than children lower on such eating behaviors.

Although cross-sectional research finds that obesogenic eating behaviors correlate with children’s weight status in the expected direction (e.g., Jansen et al., 2012, Sleddens et al., 2008, Viana et al., 2008, Webber et al., 2009), apart from research on infants (Quah et al., 2015, van Jaarsveld et al., 2014, van Jaarsveld et al., 2011), longitudinal evidence in children is both sparse and inconsistent. In the preschool years, reports chronicle no prospective associations (Bergmeier et al., 2014, Mallan et al., 2014), apart from lower satiety responsiveness forecasting higher BMI in one study (Mallan et al., 2014). In middle childhood, higher weight is predicted by some eating behaviors (Derks et al., 2018, Parkinson et al., 2010, Steinsbekk and Wichstrøm, 2015), but studies also report the opposite order of effects (i.e., increased weight predicting eating behaviors) (Bjørklund et al., 2018, Derks et al., 2018, Steinsbekk et al., 2017, Steinsbekk and Wichstrøm, 2015). Bidirectionality between eating behaviors and BMI has been reported in three of the above-mentioned studies, one examining infants (van Jaarsveld et al., 2011) and two investigating middle childhood (Derks et al., 2018, Steinsbekk and Wichstrøm, 2015). A recent review and *meta*-analysis concluded that although there is preliminary support for the hypothesis that obesogenic eating behaviors in children constitute a risk of excess weight gain, existing evidence remains weak due to a lack of longitudinal studies examining bidirectionality (Kininmonth et al., 2021).

Regardless of their conflicting nature, the above observational findings may be due to a range of unmeasured confounding. For example, twin studies show moderate to high heritability for most eating behaviors (Carnell et al., 2008, Dubois et al., 2013, Llewellyn et al., 2010) and emerging evidence suggests that eating behaviors in part mediate the effects of genes on BMI (Silventoinen & Konttinen, 2020). However, recent methodological advancements in within-person analyses, using study participants as their own controls (Berry and Willoughby, 2017, Curran and Bauer, 2011, Hamaker et al., 2015), disentangle within- and between person effects and account for confounders that do not change their value over time (e.g. genes common to both eating behaviors and BMI) even though their impact may change over time (Allison, 2009, Bollen and Brand, 2010). Studies applying such within-person analyses are needed in order to better understand the relation between childhood eating behaviors and BMI (Kininmonth et al., 2021).

Moreover, as children approach later childhood and adolescence, with increasing autonomy, they are expected to take more responsibility for their own eating, and parental impact (e.g., efforts to control intake) diminishes accordingly. Thus, the eating behaviors of older children and adolescents may be even more important for their weight development than those of younger children. Therefore, findings related to the eating behaviors of infants and young children cannot be generalized to older children and adolescents. Yet, prospective studies in late childhood and adolescence are completely lacking.

Following a community sample with biennial assessments from age 6 to 14 years and using a within-person analysis, we investigated the prospective associations between changes in eating behaviors and changes in BMI. We hypothesize a bidirectional relationship between eating behaviors and BMI: 1) increased levels of alleged obesogenic eating behaviors predict increases in BMI; and 2) increased BMI in children predicts increases in obesogenic eating behaviors.

## 2 Methods

### 2.1 Participants and procedure

**Fig. 1:**
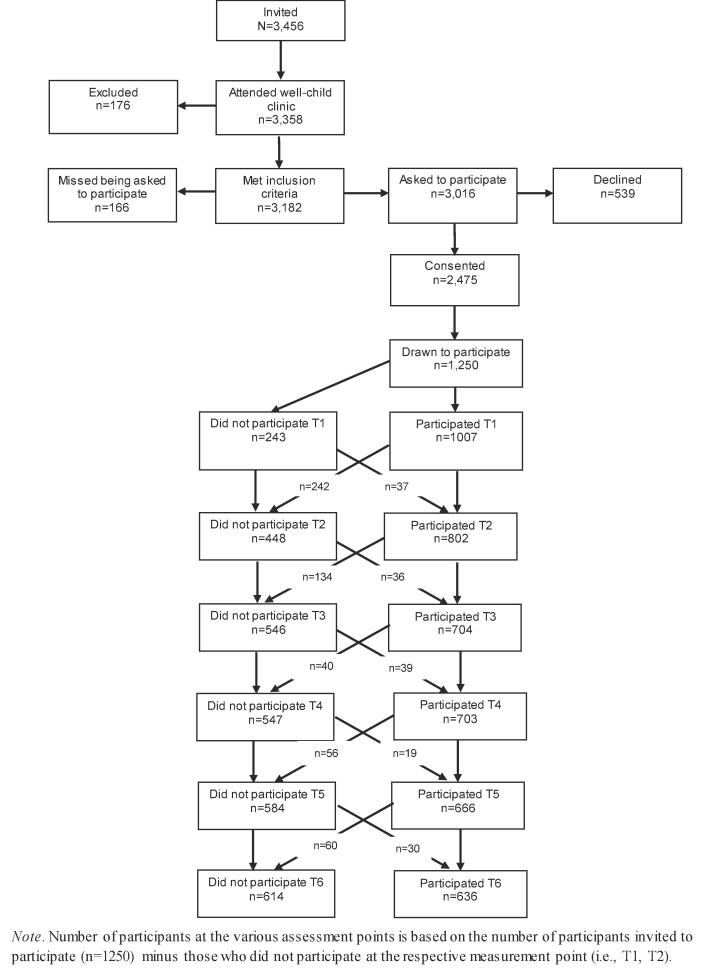
Recruitment and follow-up.

The present study is embedded in the Trondheim Early Secure Study (TESS) (Steinsbekk & Wichstrøm, 2018), a prospective on-going cohort study on children’s development. All children born in Trondheim, Norway in 2003 and 2004 (N = 3,456), and their parents, were invited to participate in TESS, which had an initial aim to examine mental health. For that reason, the invitation letter also included the Strengths and Difficulties Questionnaire (SDQ) (Goodman, 1997). When attending the national routine health check-up at age 4, which almost all children in the two cohorts did (97.2%), parents brought the completed SDQ and a health nurse obtained the parents’ written consent to participate (n = 2,475, 5.2% of eligible parents were missed being asked) (Fig. 1). Of the consenting families, children with higher SDQ scores (i.e., more problems) were oversampled to increase sample variability and thus power. This was accomplished by allocating children to four strata according to their SDQ scores (cut-offs: 0–4, 5–8, 9–11, and 12–40), and their probability of selection increased with increasing SDQ scores (0.37, 0.48, 0.70, and 0.89 in the four strata, respectively). Importantly, this oversampling was accounted for in the statistical analyses. Because of the time- and resource demanding in depth assessment of the TESS participants, only 1,250 families were drawn to participate based on the procedure described above. The study was approved by The Regional Committee for Medical and Health Research Ethics, Mid-Norway.

Children and their parents later visited the university clinic for testing and observation (2007–2008) and retesting took place when they were 6 (2009–2010), 8 (2011–2012), 10 (2013–2014), 12 (2015–2016) and 14 (2017–2018) years old. Please note that every data wave takes two years to complete (Fig. 1) because participants represent two age cohorts (i.e., born in 2003 and 2004, respectively). Table 1 presents participants’ baseline characteristics. The sample was comparable with the Norwegian parent population in terms of parents’ level of education (Statistics Norway, 2012) and children’s BMI (Juliusson et al., 2013). Eating behaviors were measured from age 6 and onwards, the current study is therefore based on data collected at age 6 (n = 797, M_age_ = 6.72 years, SD = 0.17), age 8 (n = 699, M_age_ = 8.80 years, SD = 0.24), age 10 (n = 702, M_age_ = 10.51 years, SD = 0.17), age 12 (n = 666, M_age_ = 12.49 years, SD = 0.15) and age 14 (n = 636, M_age_ = 14.33 years, SD = 0.59).

**Table 1:** Sample characteristics at enrollment (age 4).

Characteristic		%
Gender of child	Male	49.1
	Female	50.9

Gender of parent informant	Male	15.2
	Female	84.8

Ethnic origin of biological mother	Norwegian	93.0
	Western Countries	2.7
	Other Countries	4.3

Ethnic origin of biological father	Norwegian	91.0
	Western Countries	5.8
	Other Countries	3.2

Childcare	Official daycare centre	95.0
	Other	5.0

Biological parents’ marital status	Married	56.3
	Cohabitating >6 months	32.6
	Separated	1.7
	Divorced	6.8
	Widowed	0.2
	Cohabitating < 6 months	1.1
	Never lived together	1.3

Informant parent’s occupational level	Leader	5.7
	Professional, higher level	25.7
	Professional, lower level	39.0
	Formally skilled worker	26.0
	Farmer/fisherman	0.5
	Unskilled worker	3.1

Parent’s highest completed education	Did not complete junior high school	0
	Junior high school (10th grade)	0.6
	Some education after junior high school	6.1
	Senior high school (13th grade)	17.3
	Some education after senior high school	3.4
	Some college or university education	7.6
	Bachelor degree	6.2
	College degree (3–4 years study)	33.6
	Master degree or similar	20.3
	PhD completed or ongoing	4.4

Households’ gross annual income	0 – 225′ NOK (0 – 40′ USD)	3.3
	225′ – 525′ NOK (40′ – 94′ USD)	18.4
	525′ – 900′ NOK (94′- 161′ USD)	51.6
	900′ + NOK (161′+ USD)	26.7

Attrition according to each study variable was examined in SPSS version 25 using logistic regression analyses. The results from these bivariate analyses showed that attrition was selective according to the following variables: Age 10: BMI at age 8 (OR = 0.84 (95% CI, 0.73, 0.97, *p* = 0.016); Age 12: Food responsiveness (OR = 0.55 (95% CI, 0.35, 0.88, *p* = 0.012) and emotional overeating (OR = 0.40 (95% CI, 0.24, 0.68, *p* ≤ 0.001) at age 10; Age 14: BMI at age 8 (OR = 1.10 (95% CI, 1.00, 1.22, *p* = 0.047) and age 10 (OR = 1.10 (95% CI, 1.01, 1.19, *p* = 0.025); emotional overeating at age 10 (OR = 1.66 (95% CI, 1.08, 2.55, *p =* 0.020) and age 12 (OR = 1.77 (95% CI, 1.11, 2.87, *p* = 0.020). Please note that multivariate analyses revealed that the combined effects were miniscule (Age 12: Nagelkerke proxy R^2^ = 0.011, Cox & Snell = 0.005; Age 14: Nagelkerke proxy R^2^ = 0.024, Cox & Snell = 0.012).

### 2.2 Measures

#### 2.2.1 Eating behaviors

The Norwegian version of the parent-reported Children’s Eating Behaviour Questionnaire (CEBQ) (Wardle et al., 2001) was used to capture eating behaviors at ages 6–14, and was typically completed by mothers (see Table 1). The following subscales were included: Food Responsiveness (range of internal consistency for age 6 to 14: α = 0.65–0.71; 5 items, e.g. “My child is always asking for food”); Enjoyment of Food (α = 0.81–0.84; 4 items, e.g. “My child loves food”); Emotional Overeating (α = 0.75–0.80; 4 items, e.g. “My child eats more when s/he is anxious”); Emotional undereating (α = 0.75–0.84; 4 items, e.g. “My child eats less when s/he is upset”); Satiety Responsiveness (α = 0.70–0.74; 5 items, e.g. “My child gets full before his/her meal is finished”); Slowness in Eating (α = 0.60–0.72; 4 items, e.g. “My child takes more than 30 min to finish a meal”); and Food Fussiness (α = 0.89–0.90; 6 items, e.g. “My child refuses new foods at first”). The CEBQ has demonstrated good test–retest reliability (Wardle et al., 2001) and has been validated against objective measures of eating behaviors (Carnell & Wardle, 2007).

#### 2.2.2 Child BMI.

Digital scales were used to assess height (Heightronic digital stadiometer: QuickMedical, Model 235 A) and weight (Tanita BC420MA; adjusting 0.5 kg for indoor clothing). Based on these measures, BMI was calculated (Cole et al., 1998). Several studies have shown that due to lower within-child variability (Berkey and Colditz, 2007, Cole et al., 2005), BMI z-scores (e.g., Barlow et al., 2020) and BMI percentiles (e.g., Kakinami et al., 2014) are less suitable metrics in longitudinal studies compared to BMI. To preserve variability and thus statistical power, we use BMI, which is recommended in longitudinal analyses of change (Berkey and Colditz, 2007, Cole et al., 2005).

### 2.3 Statistical analyses

All analyses were performed in Mplus version 7.4 (Muthèn & Muthèn, 1998–2015) using a robust maximum likelihood estimator and probabilty weights, thus accounting for the oversampling procedure. The probabilty weights were proportional to the number of children in the population in a specific stratum divided by the number of participating children in that stratum. Missing data were handled using a Full Information Maximum Likelihood (FIML) procedure.

#### 2.3.1 Choice of statistical model: An overview

**Fig. 2:**
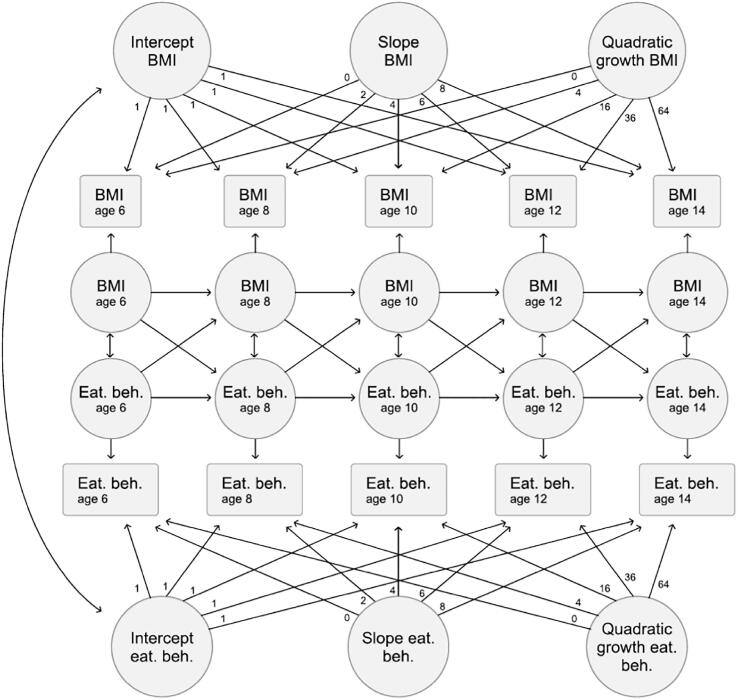
An illustration of the autoregressive latent trajectory model with structured residuals (ALT-SR). As shown, the model disaggregates the between-person association between intercepts from the within-person cross-lagged associations between BMI and eating behavior over time. BMI = Body mass index; Eat.beh. = Eating behavior.

As BMI, and possibly also eating behaviors, are expected to change with age, the relation between eating behaviors and BMI in children was examined using an autoregressive latent trajectory model with structured residuals (ALT-SR; Fig. 2). ALT-SR is a within-person analysis that allows people to be characterized by their own growth trajectory over time (Berry and Willoughby, 2017, Hamaker et al., 2015). The within-person component of the model allows each person to have his/her own time-specific deviation from his/her own *trajectory* so that systematic aspects of the phenomena of interest over time (i.e., eating behavior and BMI, in our case) are detrended (Berry & Willoughby, 2017). Consequently, the growth models of the phenomena studied represent the systematic, stable components over time, whereas the structured residuals capture the time-specific variations that remains (i.e., deviations from the child’s BMI trajectory or eating behavior trajectory, in our study). Such within-person (i.e., fixed effects) analyses implicitly adjusts for unmeasured time-invariant confounding, irrespective of whether it is known or not (Allison, 2009, Gunasekara et al., 2014, Usami et al., 2019). For more details, see Supplemental Material.

#### 2.3.2 Model fitting of growth curves

Because it is viable that increases in BMI (Boyer et al., 2015, Martins et al., 2010) and possibly also eating behaviors could be curvilinear, we first estimated their best-fitting growth trajectories using latent growth curve modelling. Intercepts were set at the start of the trajectories of BMI and eating behavior, and the slopes represented yearly changes in BMI and eating behaviors. We henceforth tested whether no, linear, or quadratic growth fitted the data best using the Satorra-Bentler scaled chi square test (Satorra & Bentler, 2001).

#### 2.3.3 ALT-SR

Based on the results from the above trajectory model fitting procedure (see Supplemental Material, Table S2), the full ALT-SR was then estimated. Latent variables loading separately on eating behaviors at each time point and BMI at each time point were created, which then effectively capture time-specific changes from the participant’s own mean and expected trajectory. Because of the high number of parameters to be estimated relative to the number of children, it was not possible to include all seven eating behaviors and BMI in one model. Separate models for each of the eating behaviors were therefore created (i.e., seven models in total). In each of these models, the changes in BMI at ages 8, 10, 12 and 14 were regressed on changes in eating behaviors at ages 6, 8, 10 and 12, respectively. To take potential bi-directionality into account, changes in eating behaviors at ages 8, 10, 12 and 14 were regressed on changes in BMI at ages 6, 8, 10 and 12, respectively. We also controlled for changes in eating behaviors and BMI two years prior (autoregressions).

## 3 Results

Descriptive statistics are shown in Table 2. As expected, higher scores for food responsiveness, emotional overeating and enjoyment of food were correlated with higher BMI at all time points, whereas higher scores for the other eating behaviors (satiety responsiveness in particular) tended to be associated with lower BMI (Table 3). Cross-sectional correlations between eating behaviors can be found in the Supplemental Material (Table S1). The results of the model fitting procedure (Table S2, Supplemental Material) revealed that a quadratic model fitted the data better than a linear model for BMI, emotional overeating, enjoyment of food, satiety responsiveness, slowness in eating and emotional undereating; thus, the quadratic growth models were included in the ALT-SR for these variables. For food responsiveness and food fussiness, the linear models were retained. The results of the ALT-SR revealed that change in eating behaviors did not predict change in BMI at any time point (Table 4, Table 5). However, evidence for the opposite direction of influence was found: Deviations from one’s expected mean level and growth in BMI predicted changes in several eating behaviors at different ages. Specifically, increases in BMI predicted more food responsiveness (B ranging from 0.03 to 0.14; 95% CI ranging from 0.01 to 0.08 to 0.05–0.20) and emotional overeating (B ranging from 0.03 to 0.06; 95% CI ranging from 0.01 to 0.02 to 0.05–0.12) at all time points, as well as greater enjoyment of food from age 8 to age 10 (B = 0.05; 95% CI: 0.02, 0.09) and from age 10 to age 12 (B=0.03; 95% CI: 0.01, 0.04). Furthermore, increases in BMI predicted decreases in satiety responsiveness at three time points (B_age8_ = −0.09; 95% CI: −0.14, −0.05; B_age10_ = −0.08; 95% CI: −0.12, −0.05; B_age12_ = −0.03; 95% CI: −0.05, −0.01), and emotional undereating, the latter from 12 to 14 years exclusively (B = −0.03; 95% CI: −0.06, −0.01).

**Table 2:** Descriptives for all study variables and the proportion of participants categorized as normal weight and overweight/obese, respectively.

	Age 6		Age 8		Age 10		Age 12		Age 14	
Study variable	Mean (SD)	Min/max	Mean (SD)	Min/max	Mean (SD)	Min/max	Mean (SD)	Min/max	Mean (SD)	Min/max
Body Mass Index	15.63 (1.49)	12.10/27.47	16.68 (1.98)	12.73/30.61	17.64 (2.53)	12.83/36.71	18.93 (2.75)	13.29/33.44	20.44 (3.0)	14.21/39.66
Food responsiveness	1.90 (0.47)	1.00/4.20	1.87 (0.48)	1.00/4.60	1.89 (0.52)	1.00/4.20	1.85 (0.51)	1.00/4.60	1.81 (0.48)	1.00/4.80
Emotional overeating	1.43 (0.44)	1.00/3.00	1.44 (0.46)	1.00/4.00	1.47 (0.49)	1.00/3.50	1.49 (0.50)	1.00/4.00	1.62 (0.54)	1.00/4.50
Enjoyment of food	3.45 (0.56)	1.75/5.00	3.50 (0.56)	1.50/5.00	3.58 (0.59)	1.50/5.00	3.59 (0.60)	1.50/5.00	3.60 (0.62)	1.25/5.00
Satiety responsiveness	2.92 (0.50)	1.20/4.20	2.80 (0.53)	1.20/4.40	2.74 (0.56)	1.00/4.40	2.63 (0.56)	1.00/4.40	2.61 (0.56)	1.00/4.80
Slowness in eating	2.55 (0.63)	1.00/4.75	2.41 (0.61)	1.00/4.75	2.36 (0.60)	1.00/4.50	2.25 (0.58)	1.00/4.50	2.24 (0.55)	1.00/4.25
Emotional undereating	2.63 (0.76)	1.00/4.75	2.48 (0.74)	1.00/4.50	2.39 (0.76)	1.00/4.75	2.22 (0.79)	1.00/4.50	2.21 (0.79)	1.00/4.25
Food fussiness	2.76 (0.73)	1.00/4.83	2.66 (0.74)	1.00/4.83	2.59 (0.76)	1.00/4.83	2.44 (0.74)	1.00/4.67	2.42 (0.74)	1.00/4.83

	NW(%)	OW/OB(%)	NW(%)	OW/OB(%)	NW(%)	OW/OB(%)	NW(%)	OW/OB(%)	NW(%)	OW/OB(%)
Percentage of children with NW^a^ and OW/OB^b^	96.0	4.0	93.3	6.7	92.5	7.5	91.6	8.4	89.0	11.0

*Note*. NW = Normal weight; OW/OB = Overweight/obesity. ^a^ It should be noted that underweight was not calculated and is thus included in the NW category; ^b^ According to the criteria of the International Obesity Task Force (IOTF).

**Table 3:** Bivariate correlations between eating behaviors and BMI.

	BMI age 6	BMI age 8	BMI age 10	BMI age 12	BMI age 14
**Eating behaviors age 6**					
FR	0.31***	0.26***	0.23***	0.15**	0.14**
EOE	0.20***	0.15***	0.16**	0.14**	0.12*
EF	0.20***	0.18***	0.15***	0.12**	0.12**
SR	−0.33***	−0.27***	−0.22***	−0.21***	−0.20***
SE	−0.13**	−0.13**	−0.13***	−0.09*	−0.06
EUE	−0.02	−0.04	−0.002	−0.01	0.002
FF	−0.14***	−0.09*	−0.09*	−0.11*	−0.09*

**Eating behaviors age 8**					
FR	0.31***	0.37***	0.29***	0.29***	0.24***
EOE	0.14**	0.19***	0.17**	0.17**	0.14**
EF	0.20***	0.21***	0.14**	0.17***	0.14**
SR	−0.38***	−0.36***	−0.26***	−0.25***	−0.22***
SE	−0.09*	−0.10*	−0.10*	−0.05	−0.07
EUE	−0.12**	−0.11**	−0.09*	−0.09	−0.05
FF	−0.09*	−0.10**	−0.07	−0.11*	−0.09

**Eating behaviors age 10**					
FR	0.22***	0.40***	0.38***	0.40***	0.32***
EOE	0.18**	0.23***	0.24***	0.24***	0.21***
EF	0.20***	0.21***	0.18***	0.17***	0.13**
SR	−0.38***	−0.40***	−0.34***	−0.35***	−0.29***
SE	−0.08	−0.07	−0.06	−0.02	−0.04
EUE	−0.04	−0.05	−0.01	−0.01	−0.03
FF	−0.03	−0.03	−0.03	−0.05	−0.07

**Eating behaviors age 12**					
FR	0.15**	0.26***	0.25***	0.32***	0.27***
EOE	0.10*	0.19**	0.18***	0.23***	0.20***
EF	0.14**	0.16***	0.16***	0.15***	0.14**
SR	−0.33***	−0.34***	−0.29***	−0.30***	−0.27***
SE	−0.07	−0.06	−0.03	0.01	−0.01
EUE	−0.09*	−0.08	−0.05	−0.05	−0.06
FF	−0.05	−0.06	−0.06	−0.06	−0.08

**Eating behaviors age 14**					
FR	0.09	0.19***	0.15**	0.21***	0.20***
EOE	0.07	0.14**	0.11*	0.18***	0.17**
EF	0.10*	0.16***	0.13**	0.13**	0.13**
SR	−0.16**	−0.19***	−0.15***	−0.17***	−0.16***
SE	−0.06	−0.05	−0.06	−0.05	−0.08
EUE	−0.10*	−0.09*	−0.12*	−0.13**	−0.10*
FF	−0.06	−0.10*	−0.08*	−0.10**	−0.09*

*Note*. BMI = Child body mass index; FR = Food responsiveness; EOE = Emotional overeating; EF = Enjoyment of food; SR = Satiety responsiveness; SE = Slowness in eating; EUE = Emotional undereating; FF = Food fussiness; *p < 0.05; **p < 0.01; ***p < 0.001.

**Table 4:** Bidirectional paths between eating behaviors and BMI – food responsiveness, emotional overeating and enjoyment of food.

	Body Mass Index				Food responsiveness a				Emotional overeating ^b^				Enjoyment of food ^c^			
	*B (S.E.)*	95% CI	*β*	*p*	*B (S.E.)*	95% CI	*β*	*p*	*B (S.E.)*	95% CI	*β*	*p*	*B (S.E.)*	95% CI	*β*	*p*
**Age 8**																
BMI age 6	1.22 (0.05)	1.12, 1.32	0.78	<0.001	0.14 (0.03)	0.08, 0.20	0.47	<0.001	0.06 (0.03)	0.01, 0.12	0.24	0.02	0.04 (0.02)	−0.003, 0.09	0.14	0.07
FR age 6	−0.20 (0.22)	−0.62, 0.22	−0.03	0.35	0.08 (0.09)	−0.09, 0.25	0.07	0.34								
EOE age 6	−0.13 (0.26)	−0.65, 0.38	−0.02	0.61					0.11 (0.07)	−0.04, 0.25	0.10	0.16				
EF age 6	0.06 (0.17)	−0.27, 0.39	0.01	0.74									0.32 (0.06)	0.20, 0.45	0.30	<0.001

**Age 10**																
BMI age 8	1.11 (0.05)	1.01, 1.21	0.86	<0.001	0.11 (0.02)	0.08, 0.13	0.50	<0.001	0.06 (0.02)	0.02, 0.09	0.29	0.002	0.05 (0.02)	0.02, 0.09	0.26	0.006
FR age 8	0.07 (0.20)	−0.34. 0.47	0.01	0.74	0.22 (0.08)	0.06, 0.38	0.20	0.008								
EOE age 8	−0.02 (0.34)	−0.68, 0.64	−0.002	0.96					0.27 (0.08)	0.12, 0.42	0.25	<0.001				
EF age 8	−0.25 (0.24)	−0.71, 0.22	−0.04	0.30									0.26 (0.07)	0.13, 0.40	0.26	<0.001

**Age 12**																
BMI age 10	0.96 (0.07)	0.82, 1.10	0.92	<0.001	0.04 (0.01)	0.02, 0.07	0.30	0.001	0.03 (0.01)	0.01, 0.06	0.24	0.007	0.03 (0.01)	0.01, 0.04	0.15	0.005
FR age 10	0.41 (0.39)	−0.36, 1.18	0.07	0.29	0.27 (0.08)	0.12, 0.42	0.29	<0.001								
EOE age 10	0.68 (0.61)	−0.51, 1.87	0.10	0.26					0.18 (0.13)	−0.07, 0.43	0.19	0.16				
EF age 10	0.49 (0.50)	−0.43, 1.41	0.08	0.30									0.34 (0.08)	0.17, 0.50	0.34	<0.001

**Age 14**																
BMI age 12	0.97 (0.04)	0.89, 1.04	0.89	<0.001	0.03 (0.01)	0.01, 0.05	0.22	<0.001	0.03 (0.01)	0.01, 0.05	0.18	0.02	0.02 (0.01)	−0.01, 0.04	0.09	0.19
FR age 12	0.12 (0.22)	−0.32, 0.55	0.02	0.60	0.27 (0.08)	0.11, 0.43	0.28	0.001								
EOE age 12	0.12 (0.20)	−0.28, 0.52	0.01	0.57					0.28 (0.12)	0.06, 0.51	0.24	0.01				
EF age 12	0.02 (0.19)	−0.36, 0.40	0.003	0.92									0.34 (0.07)	0.20, 0.47	0.30	<0.001

*Note.* BMI = Body mass index; FR = Food responsiveness; EOE = Emotional overeating; EF = Enjoyment of food.

a Model fit indices for FR model: χ^2^ = 86.55 (*p* <.001); CFI = 0.971; TLI = 0.960; RMSEA = 0.05 (90% CI: 0.03, 0.06); SRMR = 0.08; ^b^Model fit indices for EOE model: χ^2^ = 167.39 (*p* <.001); CFI = 0.923; TLI = 0.892; RMSEA = 0.07 (90% CI: 0.06, 0.08); SRMR = 0.09; ^c^Model fit indices for EF model: χ^2^ = 145.167 (*p* <.001); CFI = 0.945; TLI = 0.923; RMSEA = 0.07 (90% CI: 0.06, 0.08); SRMR = 0.08.

**Table 5:** Bidirectional paths between eating behaviors and BMI – satiety responsiveness, slowness in eating, emotional undereating and fussiness.

	**Body Mass Index**				**Satiety responsiveness**^a^				**Slowness in eating ^b^**				**Emotional undereating ^c^**				**Food fussiness ^d^**			
	*B (S.E.)*	95% CI	*β*	*p*	*B (S.E.)*	95% CI	*β*	*p*	*B (S.E.)*	95% CI	*β*	*p*	*B (S.E.)*	95% CI	*β*	*p*	*B (S.E.)*	95% CI	*β*	*p*
**Age 8**																				
BMI 6	1.22 (0.05)	1.12, 1.32	0.78	<0.001	−0.09 (0.03)	−0.14, −0.05	−0.29	<0.001	−0.01 (0.03)	−0.07, 0.05	−0.02	0.84	−0.04(0.03)	−0.11, 0.02	−0.10	0.18	−0.01 (0.03)	−0.08, 0.06	−0.03	0.76
SR 6	0.14 (0.18)	−0.20, 0.49	0.03	0.41	0.24 (0.07)	0.10, 0.38	0.22	0.001												
SE 6	0.06 (0.11)	−0.15, 0.27	0.01	0.57					0.29 (0.08)	0.14, 0.44	0.28	<0.001								
EUE 6	−0.13 (0.09)	−0.30, 0.04	−0.04	0.14									0.19 (0.06)	0.07, 0.31	0.18	0.002				
FF 6	0.11 (0.15)	−0.18, 0.41	0.02	0.45													0.24 (0.06)	0.12, 0.36	0.23	<0.001

**Age 10**																				
BMI 8	1.11 (0.05)	1.01, 1.21	0.86	<0.001	−0.08 (0.02)	−0.12, −0.05	−0.37	<0.001	0.01 (0.02)	−0.02, 0.04	0.04	0.62	−0.01(0.03)	−0.05, 0.04	−0.02	0.82	0.01 (0.02)	−0.03, 0.06	0.06	0.53
SR 8	0.44 (0.37)	−0.29, 1.17	0.07	0.23	0.31 (0.07)	0.18, 0.44	0.28	<0.001												
SE 8	−0.23 (0.18)	−0.58, 0.12	−0.04	0.20					0.28 (0.06)	0.16, 0.40	0.28	<0.001								
EUE 8	0.003 (0.17)	−0.33, 0.33	0.001	0.98									0.15 (0.07)	0.01, 0.30	0.15	0.04				
FF 8	−0.18 (0.23)	−0.63, 0.27	−0.03	0.44													0.39 (0.08)	0.24, 0.54	0.37	<0.001

**Age 12**																				
BMI 10	0.96 (0.07)	0.82, 1.10	0.92	<0.001	−0.03 (0.01)	−0.05, −0.01	−0.17	0.01	0.004(0.01)	−0.02, 0.02	0.03	0.66	−0.01(0.02)	−0.04, 0.02	−0.04	0.57	−0.01 (0.01)	−0.03, 0.02	−0.03	0.66
SR 10	−0.72 (0.52)	−1.74, 0.29	−0.12	0.16	0.36 (0.09)	0.19, 0.53	0.38	<0.001												
SE 10	0.34 (0.26)	−0.16, 0.85	0.06	0.18					0.31 (0.07)	0.18, 0.44	0.30	<0.001								
EUE 10	−0.07 (0.24)	−0.53, 0.40	−0.01	0.78									0.18 (0.08)	0.01, 0.34	0.18	0.04				
FF 10	0.13 (0.39)	−0.63, 0.88	0.02	0.75													0.44 (0.07)	0.31, 0.56	0.42	<0.001

**Age 14**																				
BMI 12	0.97 (0.04)	0.89, 1.04	0.89	<0.001	−0.004 (0.01)	−0.02, 0.02	−0.02	0.71	−0.01 (0.01)	−0.03, 0.01	−0.04	0.46	−0.03(0.01)	−0.06. −0.01	−0.14	0.03	0.003 (0.01)	−0.02, 0.03	0.02	0.82
SR 12	0.02 (0.19)	−0.36, 0.39	0.003	0.93	0.44 (0.05)	0.34, 0.54	0.42	<0.001												
SE 12	−0.10 (0.18)	−0.46, 0.26	−0.02	0.58					0.31 (0.06)	0.19, 0.43	0.32	<0.001								
EUE 12	−0.06 (0.11)	−0.27, 0.17	−0.01	0.63									0.27 (0.06)	0.15, 0.39	0.25	<0.001				
FF 12	−0.19 (0.20)	−0.58, 0.19	−0.03	0.33													0.46 (0.06)	0.34, 0.58	0.43	<0.001

*Note.* BMI = Body mass index; SR = Satiety responsiveness; SE = Slowness in eating; EUE = Emotional undereating; FF = Food fussiness.

a Model fit indices for SR model: χ^2^ = 145.66 (*p* < 0.001); CFI = 0.945; TLI = 0.923; RMSEA = 0.07 (90% CI: 0.06, 0.08); SRMR = 0.08; ^b^Model fit indices for SE model: χ^2^ = 156.02 (*p* < 0.001); CFI = 0.937; TLI = 0.911; RMSEA = 0.07 (90% CI: 0.06, 0.08); SRMR = 0.10; ^c^Model fit indices for EUE model: χ^2^ = 131.32 (*p* < 0.001); CFI = 0.944; TLI = 0.921; RMSEA = 0.06 (90% CI: 0.05, 0.07); SRMR = 0.08; ^d^Model fit indices for FF model: χ^2^ = 147.84 (*p* < 0.001); CFI = 0.953; TLI = 0.936; RMSEA = 0.07 (90% CI: 0.06, 0.08); SRMR = 0.08.

## 4 Discussion

We examined the relation between change in eating behaviors and BMI from childhood to adolescence, net of time-invariant confounding. To our knowledge, no prior study has investigated this association with several repeated measurements, covering the years from early school age to adolescence. In contrast to what we hypothesized, eating behaviors did not predict increased or decreased BMI at any time point. Rather, the results showed the opposite direction of effects: Changes in BMI predicted changes in eating behaviors, over and beyond each individual’s own expected developmental trajectory. This pattern was seen for all eating behaviors, except for eating speed and food fussiness.

The finding that eating behaviors did not predict BMI runs counter to common beliefs and contrasts with findings in infancy (Quah et al., 2015, van Jaarsveld et al., 2014, van Jaarsveld et al., 2011) and middle childhood (Parkinson et al., 2010, Steinsbekk and Wichstrøm, 2015) that obesogenic eating behaviors predict higher weight prospectively. Yet, our null results concur with a Dutch study reporting that eating behaviors do not predict higher weight from 4 to 10 years of age, with the exception of emotional overeating (Derks et al., 2018). Instead, we found that higher BMI predicted more food responsiveness and emotional overeating at all time points, and more enjoyment of food and less satiety responsiveness at most timepoints, which also corresponds to the findings of Derks et al. (Derks et al., 2018). In addition, our results are in line with previous studies finding evidence of higher weight predicting more food responsiveness (Bjørklund et al., 2018, Steinsbekk and Wichstrøm, 2015) and lower satiety responsiveness (Steinsbekk & Wichstrøm, 2015). To our knowledge, the current study is the first to establish these prospective associations in older children and adolescents. It is possible that the impact of eating behaviors on weight development differs across childhood, potentially being important to the development of weight in infants and toddlers only. However, from school age and onwards, it may be that weight status drives the development of eating behaviors, and not the other way around.

One interpretation of our findings is that children’s appetite increases according to their body’s needs for growth, maintenance, and increased work of a heavier body. Furthermore, pubertal change represents a universal characteristic of adolescence involving rapid physical changes and growth (Petersen, 1988). Because there is no consistent effect of BMI on the intensity and duration of physical activity in children and adolescents (measured by accelerometer) (Wiersma et al., 2020), the same movement with a heavier body will cause increased energy needs and thus increased food intake, which in turn may be observed and reported by parents as increased obesogenic eating. If this is the case, parental reports of eating behaviors that are presumed to be obesogenic may indeed accurately reflect high or increased consumption, but not excess consumption beyond energy needs.

Biological mechanisms may also be important in explaining our findings. One possibility is that higher BMI upregulates appetite by an increased set point for energy balance, so that the body tries to maintain the current weight status (Friedman and Halaas, 1998, Keesey and Hirvonen, 1997). Considering that the homeostatic appetite regulation system defends well against energy deficit, but is far less effective in defense of energy excess (Harrold et al., 2012), it may be plausible that the higher levels of obesogenic eating behaviors seen in our study reflect an elevated set point driven by higher weight status, leading to increased energy intake. Another possible biological mechanism is impaired leptin signaling, leading to decreased satiety signals and consequently excess eating (Kelesidis et al., 2010, Myers et al., 2008). Such decreased leptin sensitivity is most often seen in adults with obesity, but it is an open question for future research whether such a hypothetical mechanism may be operational across different BMI- and age groups.

The finding that eating behaviors did not predict BMI in the current study may in part explain why most prevention- and treatment programs for childhood obesity have limited effects on weight outcomes (Ells et al., 2018, Hennessy et al., 2019), particularly in middle childhood (Ells et al., 2018). As already noted, interventions targeting children’s eating behaviors include promotion of parental feeding practices that encourages infants’ self-regulation of eating (Daniels et al., 2009, Daniels et al., 2013, Daniels et al., 2015, Harris et al., 2020), programs directly focusing on children’s eating behaviors (Boutelle et al., 2020, Boutelle et al., 2014, Johnson, 2000), or general self-regulation skills as a mean to improve food-specific self-regulation (Lumeng et al., 2017, Smith et al., 2015). One implication of our findings, if replicated, could be that prevention- and intervention programs targeting eating behaviors to change weight outcomes may need to focus on children younger than age 6. Furthermore, it is possible that eating behaviors could be important targets in other samples, including populations at higher genetic risk for obesity, those who show an unhealthy increase in weight as well as clinical samples. These are issues that should be addressed by future research.

### 4.1 Strengths and limitations

The present study has many strengths, including a large community sample with repeated measurements spanning over several years, and the use of an analytical technique that allowed us to separate within- and between-person variance and thereby account for all unmeasured time-invariant confounders. One important stable third-variable factor could be common underlying genes, affecting both BMI and eating behaviors (Silventoinen & Konttinen, 2020). Although time-invariant third-variable effects were adjusted for, *time-varying* confounders, including time-varying impacts of time-invariant factors (e.g., genetic innovations and altered impact by age), might still have influenced the link between BMI and future change in eating behaviors. Examples of such time-varying factors are physical activity, mood/state effects (when completing questionnaires), unstable aspects of parenting (e.g., in response to changes in family situation) and negative life events (see Supplemental Material). However, these and other time-varying factors less likely account for the *lacking* prospective association between eating behaviors and later BMI.

Reliance on parent reports of eating behaviors is a potential limitation and observational measures of eating would have been preferable. Notably though, the Children’s Eating Behavior Questionnaire (CEBQ) has been validated against observational measures of eating (Carnell & Wardle, 2007). Moreover, CEBQ is validated up to 12 years of age (Wardle et al., 2001), but in order to avoid confounding age-related changes with measurement changes, CEBQ was also used at age 14 in the current study. Although the internal reliability was lower than generally acceptable (α < 0.70) at some time points for food responsiveness and slowness in eating, it should be noted that our age-14 results do not depart from when the participants were younger, and results involving these two eating behaviors do not differ from those with higher internal consistencies – indicating that reliability issues did not produce our null-results. Another possible limitation is that, to our knowledge, no relevant cutoffs or norms concerning CEBQ exist. Therefore, little is known about how much change in CEBQ scores is needed to reflect clinically relevant changes in eating behaviors – which could be important to address in future research. Furthermore, the reliance on BMI rather than fat mass may also be seen as a limitation. However, studies show that change in BMI is highly correlated with change in fat mass objectively measured by dual-energy X-ray absorptiometry (DXA) (e.g., Kakinami et al., 2014), indicating that change in BMI is indeed a valid proxy for change in adiposity in children over time.

The present study was conducted in a country (i.e., Norway) with a relatively homogenous and well-educated population, therefore the findings may not generalize to more diverse populations. The results should be replicated in other samples, including more heterogeneous samples with regards to ethnic origin and SES, clinical samples, and samples with older adolescents. In addition, future studies should examine potential moderators, for example gender and parental eating/weight status.

### 4.2 Conclusions

Examining a community sample of 6-year-olds with biennial assessments until age 14, this study showed that changes in children’s BMI predicted changes in future eating behaviors, over and beyond what can be expected based on each individual’s own developmental trajectory. However, we found no evidence for the hypothesis that eating behaviors predict later BMI. One implication of our findings, if replicated, is that targeting obesogenic eating behaviors to change weight outcomes may be less effective in children older than age 6.

## Data Availability

The datasets generated and/or analyzed during the current study are not publicly available due to restrictions related to participant consent and because the study is still ongoing, but potential collaborators are welcome to contact the PI of the study.

## Funding

This research was funded by the Research Council of Norway [grant numbers 213793, 301446]; and the Liaison Committee between Central Norway RHA and NTNU.

### CRediT authorship contribution statement

**Oda Bjørklund:** Conceptualization, Methodology, Formal analysis, Writing – original draft, Visualization. **Lars Wichstrøm:** Conceptualization, Methodology, Formal analysis, Writing – review & editing, Supervision, Funding acquisition. **Clare Llewellyn:** Writing – review & editing. **Silje Steinsbekk:** Conceptualization, Writing – review & editing, Supervision, Project administration.

## Declaration of Competing Interest

The authors declare that they have no known competing financial interests or personal relationships that could have appeared to influence the work reported in this paper.
