# Supplementary material for: The prospective relation between eating behaviors and BMI from middle childhood to adolescence: A 5-wave community study
Source: Prev Med Rep. 2022 Apr 19;27:101795. doi: 10.1016/j.pmedr.2022.101795 (PMC9152788; doi:10.1016/j.pmedr.2022.101795)
Supplement: Supplementary data 1 [file mmc1.docx]

**Supplemental material for “The prospective relation between eating behaviors and BMI from middle childhood to adolescence: A 5-wave community study”**

**Contents:**

1. Comment: Statistical analyses – general issues
2. Table S1. Cross-sectional bivariate correlations between eating behaviors
3. Table S2. Results of model fitting of growth curves
4. Table S3. Stability in eating behaviors (bivariate between-time correlations)

**1. Comment**

**Statistical analyses – general issues**

*Time-invariant and time-varying variables.* Conceptually, variables may be categorized into two different types: 1) Time-invariant factors which are stable and do not change over the observation period, such as gender, and 2) time-varying variables that do change over time, such as life-events, friendships, schooling. However, it should be noted that time-varying factors may have stable components. For example, aspects of parenting may be both stable (e.g. parenting style) transient over time (e.g. in response to the child’s development, family situation etc.) (Murphy, Wickramaratne, & Weissman, 2010). Importantly, although the *value* of the time-invariant factor (e.g. genes) does not change over time, its impact may in fact change (e.g. epigenetics). Common methodological confounders, such as response bias, may similarly have both time-invariant (e.g., acquiescence and common method effects) and time-varying aspects (e.g., mood-of-the-day effects). Regarding the relation between eating behaviors and BMI, genetics, gender, parental BMI, parental rater bias and socioeconomic status (SES) are potential confounders and thus need to be adjusted for. Although these factors vary over time, they do have stable aspects that will be accounted for in analyses, using the ALT-SR (described in detail below), which adjust for unmeasured time-invariant confounding.

*Within- and between person variance.* When examining the relation between eating behaviors and BMI, the commonly used cross-lagged panel model (CLPM) poses the question “Will children with more presumed obesogenic eating behaviors *than other children* have higher BMI *than other children* (or vice versa), adjusted for their prior levels of these variables?” The answer (i.e. coefficients) is a mixture of within-person- and between-person effects (Berry & Willoughby, 2017; Curran & Bauer, 2011; Hamaker, Kuiper, & Grasman, 2015). However, because other (unacquainted) children’s BMI or eating behaviors cannot be part of the etiology of weight or eating in a specific child, a more relevant question to ask is: “If this child increases in presumed obesogenic eating behaviors, will BMI increase more in the future than otherwise expected?” If one is interested in answering this question, which has etiological implications, within-person analyses are needed (Berry & Willoughby, 2017; Curran & Bauer, 2011). Importantly, using the CLPM and thus mixing between-person and within-person information, may lead to invalid conclusions (Berry & Willoughby, 2017; Curran & Bauer, 2011).

*ALT-SR model*. In the perhaps most used cross-lagged within-person analysis, the random intercept cross-lagged model (Hamaker et al., 2015), deviations from one’s own mean over the observation period are used in the analysis. Using the average (i.e., random intercept) to represent the typical value of a person may be an appropriate strategy when people do not change systematically over time. However, BMI in children is expected to increase throughout childhood (Rosner, Prineas, Loggie, & Daniels, 1998), thus averaged BMI from age 6 to 14 may not be a valid representation of their typical BMI at most ages. The autoregressive latent trajectory model with structured residuals (ALT-SR) is one type of within-person analysis (Berry & Willoughby, 2017; Usami, Murayama, & Hamaker, 2019) that allows people to be characterized by their own growth trajectory over time (Hamaker et al., 2015) while using each person as his/her own control.

Because the ALT-SR adjusts for time-invariant factors, the present study did not include covariates considered to be relatively stable over time (e.g. gender, stable aspects of parental socio-economic position and BMI).

**References**

Berry, D., & Willoughby, M. T. (2017). On the Practical Interpretability of Cross-Lagged Panel Models: Rethinking a Developmental Workhorse. *Child Development, 88*(4), 1186-1206. doi:10.1111/cdev.12660

Curran, P. J., & Bauer, D. J. (2011). The Disaggregation of Within-Person and Between-Person Effects in Longitudinal Models of Change. *Annual review of psychology, 62*(1), 583-619. doi:10.1146/annurev.psych.093008.100356

Hamaker, E. L., Kuiper, R. M., & Grasman, R. (2015). A Critique of the Cross-Lagged Panel Model. *Psychological Methods, 20*(1), 102-116. doi:10.1037/a0038889

Murphy, E., Wickramaratne, P., & Weissman, M. (2010). The stability of parental bonding reports: A 20-year follow-up. *Journal of Affective Disorders, 125*(1), 307-315. doi:<https://doi.org/10.1016/j.jad.2010.01.003>

Rosner, B., Prineas, R., Loggie, J., & Daniels, S. R. (1998). Percentiles for body mass index in US children 5 to 17 years of age. *Journal of Pediatrics, 132*(2), 211-222. doi:10.1016/s0022-3476(98)70434-2

Usami, S., Murayama, K., & Hamaker, E. L. (2019). A Unified Framework of Longitudinal Models to Examine Reciprocal Relations. *Psychological Methods, 24*(5), 637-657. doi:10.1037/met0000210

**2. Table S1. Cross-sectional bivariate correlations between eating behaviors**

| **Age 6** | **1.** | **2.** | **3.** | **4.** | **5.** | **6.** | **7.** |
| --- | --- | --- | --- | --- | --- | --- | --- |
| 1. FR age 6 | - |  |  |  |  |  |  |
| 2. EOE age 6 | .56*** | - |  |  |  |  |  |
| 3. EF age 6 | .28*** | .09* | - |  |  |  |  |
| 4. SR age 6 | -.18*** | -.002 | -.51*** | - |  |  |  |
| 5. SE age 6 | .01 | -.001 | -.12** | .20*** | - |  |  |
| 6. EUE age 6 | .18*** | .35*** | -.09* | .25*** | .13** | - |  |
| 7. FF age 6 | .002 | .08 | -.54*** | .38*** | .05 | .20*** | - |
| **Age 8** | **1.** | **2.** | **3.** | **4.** | **5.** | **6.** | **7.** |
| 1. FR age 8 | - |  |  |  |  |  |  |
| 2. EOE age 8 | .52*** | - |  |  |  |  |  |
| 3. EF age 8 | .28*** | .04 | - |  |  |  |  |
| 4. SR age 8 | -.24*** | -.004 | -.51*** | - |  |  |  |
| 5. SE age 8 | .07 | .07 | -.08 | .23*** | - |  |  |
| 6. EUE age 8 | .17*** | .39*** | -.07 | .25*** | .23*** | - |  |
| 7. FF age 8 | .04 | .15*** | -.51*** | .39*** | .04 | .14** | - |
| **Age 10** | **1.** | **2.** | **3.** | **4.** | **5.** | **6.** | **7.** |
| 1. FR age 10 | - |  |  |  |  |  |  |
| 2. EOE age 10 | .61*** | - |  |  |  |  |  |
| 3. EF age 10 | .29*** | .11** | - |  |  |  |  |
| 4. SR age 10 | -.22*** | -.03 | -.49*** | - |  |  |  |
| 5. SE age 10 | .07 | .08* | -.04 | .20*** | - |  |  |
| 6. EUE age 10 | .23*** | .43*** | -.04 | .24*** | .17*** | - |  |
| 7. FF age 10 | .04 | .11** | -.52*** | .38*** | .01 | .13** | - |
| **Age 12** | **1.** | **2.** | **3.** | **4.** | **5.** | **6.** | **7.** |
| 1. FR age 12 | - |  |  |  |  |  |  |
| 2. EOE age 12 | .62*** | - |  |  |  |  |  |
| 3. EF age 12 | .29*** | .10* | - |  |  |  |  |
| 4. SR age 12 | -.12** | .05 | -.41*** | - |  |  |  |
| 5. SE age 12 | .08 | .09* | -.05 | .29*** | - |  |  |
| 6. EUE age 12 | .31*** | .51*** | .03 | .26*** | .15*** | - |  |
| 7. FF age 12 | .07 | .11* | -.44*** | .31*** | .09 | .10* | - |
| **Age 14** | **1.** | **2.** | **3.** | **4.** | **5.** | **6.** | **7.** |
| 1. FR age 14 | - |  |  |  |  |  |  |
| 2. EOE age 14 | .59*** | - |  |  |  |  |  |
| 3. EF age 14 | .26*** | .08 | - |  |  |  |  |
| 4. SR age 14 | -.06 | .15*** | -.42*** | - |  |  |  |
| 5. SE age 14 | .10* | .12** | -.09* | .27*** | - |  |  |
| 6. EUE age 14 | .27*** | .55*** | -.07 | .35*** | .24*** | - |  |
| 7. FF age 14 | -.02 | .08 | -.48*** | .35*** | .05 | .13** | - |

*Note*. FR=Food responsiveness; EOE=Emotional overeating; EF=Enjoyment of food; SR=Satiety responsiveness; SE=Slowness in eating; EUE=Emotional undereating; FF=Food fussiness; *p<.05; **p<.01; ***p<.001.

**3. Table S2. Results of model fitting of growth curves**

| Food responsiveness | χ^2^ | df | p-value | Δχ^2^ | df (diff.) | p-value | RMSEA^b^ (90% CI) | SRMR^c^ | CFI^d^ | TLI^e^ |
| --- | --- | --- | --- | --- | --- | --- | --- | --- | --- | --- |
| M1: Baseline model^a^ | 1002.40 | 10 | <.001 |  |  |  |  |  |  |  |
| **M2: Linear model** | **41.84** | **11** | **<.001** |  |  |  | **.06 (.04, .08)** | **.11** | **.969** | **.972** |
| M3: Quadratic model | 41.58 | 9 | <.001 | 1.13 | 2 | .57 | .07 (.05, .09) | .12 | .967 | .964 |
| Emotional overeating |  |  |  |  |  |  |  |  |  |  |
| M1: Baseline model^a^ | 779.52 | 10 | <.001 |  |  |  |  |  |  |  |
| M2: Linear model | 43.82 | 11 | .001 |  |  |  | .06 (.04, .08) | .07 | .957 | .961 |
| **M3: Quadratic model** | **26.24** | **9** | **.002** | **14.08** | **2** | **<.001** | **.05 (.03, .07)** | **.05** | **.978** | **.975** |
| Enjoyment of food |  |  |  |  |  |  |  |  |  |  |
| M1: Baseline model^a^ | 1234.29 | 10 | <.001 |  |  |  |  |  |  |  |
| M2: Linear model | 48.77 | 11 | <.001 |  |  |  | .07 (.05, .09) | .15 | .969 | .972 |
| **M3: Quadratic model** | **42.06** | **9** | **<.001** | **6.90** | **2** | **.032** | **.07 (.05, .09)** | **.15** | **.973** | **.970** |
| Satiety responsiveness |  |  |  |  |  |  |  |  |  |  |
| M1: Baseline model^a^ | 1127.34 | 10 | <.001 |  |  |  |  |  |  |  |
| M2: Linear model | 69.61 | 11 | <.001 |  |  |  | .08 (.06, .10) | .16 | .948 | .952 |
| **M3: Quadratic model** | **63.07** | **9** | **<.001** | **6.93** | **2** | **.031** | **.09 (.07, .11)** | **.15** | **.952** | **.946** |
| Slowness in eating |  |  |  |  |  |  |  |  |  |  |
| M1: Baseline model^a^ | 952.95 | 10 | <.001 |  |  |  |  |  |  |  |
| M2: Linear model | 90.24 | 11 | <.001 |  |  |  | .10 (.08, .11) | .17 | .916 | .924 |
| **M3: Quadratic model** | **74.83** | **9** | **.001** | **15.40** | **2** | **<.001** | **.10 (.08, .11)** | **.16** | **.930** | **.922** |
| Emotional undereating |  |  |  |  |  |  |  |  |  |  |
| M1: Baseline model^a^ | 728.64 | 10 | <.001 |  |  |  |  |  |  |  |
| M2: Linear model | 31.78 | 11 | .001 |  |  |  | .05 (.03, .07) | .08 | .971 | .974 |
| **M3: Quadratic model** | **23.80** | **9** | **.005** | **7.86** | **2** | **.020** | **.05 (.02, .07)** | **.07** | **.979** | **.977** |
| Food fussiness |  |  |  |  |  |  |  |  |  |  |
| M1: Baseline model^a^ | 1975.57 | 10 | <.001 |  |  |  |  |  |  |  |
| **M2: Linear model** | **59.99** | **11** | **<.001** |  |  |  | **.07 (.06, .09)** | **.10** | **.975** | **.977** |
| M3: Quadratic model | 59.06 | 9 | <.001 | 2.04 | 2 | .36 | .08 (.06, .10) | .10 | .975 | .972 |
| Child body mass index |  |  |  |  |  |  |  |  |  |  |
| M1: Baseline model^a^ | 478.48 | 10 | <.001 |  |  |  |  |  |  |  |
| M2: Linear model | 59.88 | 10 | <.001 |  |  |  | .08 (.06, .10) | .24 | .894 | .894 |
| **M3: Quadratic model** | **11.18** | **6** | **.082** | **34.96** | **4** | **<.001** | **.03 (<.001, .06)** | **.05** | **.989** | **.982** |

*Note*. All models are nested and compared with the next model; Δχ^2^ is corrected according to Satorra-Bentler’s procedure; preferred model in bold.

^a^ The baseline model is an unstructured model (null model/null hypothesis) assuming zero covariation between the observed variables; ^b^ Root mean square error of approximation; ^c^ Standardized root mean square residual; ^d^ Comparative fit index; ^e^ Tucker Lewis index

**4. Table S3. Stability in eating behaviors (bivariate between-time correlations)**

|  | | FR 6 | FR 8 | | FR 10 | FR 12 | FR 14 | |  | | EOE 6 | | EOE 8 | EOE 10 | EOE 12 | EOE 14 |  | EF 6 | EF 8 | EF 10 | EF 12 | EF 14 | |
| --- | --- | --- | --- | --- | --- | --- | --- | --- | --- | --- | --- | --- | --- | --- | --- | --- | --- | --- | --- | --- | --- | --- | --- |
| FR 6 | | - |  | |  |  |  | | EOE 6 | | - | |  |  |  |  | EF 6 | - |  |  |  |  | |
| FR 8 | | .65*** | - | |  |  |  | | EOE 8 | | .56*** | | - |  |  |  | EF 8 | .69*** | - |  |  |  | |
| FR 10 | | .61*** | .67*** | | - |  |  | | EOE 10 | | .58*** | | .66*** | - |  |  | EF 10 | .62*** | .68*** | - |  |  | |
| FR 12 | | .52*** | .59*** | | .66*** | - |  | | EOE 12 | | .39*** | | .55*** | .58*** | - |  | EF 12 | .52*** | .58*** | .69*** | - |  | |
| FR 14 | | .45*** | .54*** | | .53*** | .63*** | - | | EOE 14 | | .42*** | | .54*** | .58*** | .60*** | - | EF 14 | .45*** | .53*** | .62*** | .64*** | - | |
|  |  |  |  |  |  |  |  |  |  |  |  |  |  |  |  |  |  |  |  |  |  |  |  |
|  | SR 6 | SR 8 | SR 10 | SR 12 | SR 14 |  | SE 6 | SE 8 | SE 10 | SE 12 | SE 14 |  | EUE 6 | EUE 8 | EUE 10 | EUE 12 | EUE 14 |  | FF 6 | FF 8 | FF 10 | FF 12 | FF 14 |
| SR 6 | - |  |  |  |  | SE 6 | - |  |  |  |  | EUE 6 | - |  |  |  |  | FF 6 | - |  |  |  |  |
| SR 8 | .64*** | - |  |  |  | SE 8 | .67*** | - |  |  |  | EUE 8 | .59*** | - |  |  |  | FF 8 | .77*** | - |  |  |  |
| SR 10 | .56*** | .67*** | - |  |  | SE 10 | .59*** | .67*** | - |  |  | EUE 10 | .51*** | .57*** | - |  |  | FF 10 | .72*** | .81*** | - |  |  |
| SR 12 | .50*** | .58*** | .66*** | - |  | SE 12 | .54*** | .58*** | .68*** | - |  | EUE 12 | .46*** | .54*** | .57*** | - |  | FF 12 | .66*** | .75*** | .81*** | - |  |
| SR14 | .41*** | .44*** | .54*** | .65*** | - | SE14 | .42*** | .50*** | .58*** | .64*** | - | EUE 14 | .39*** | .48*** | .48*** | .59*** | - | FF 14 | .60*** | .68*** | .74*** | .79*** | - |

*Note.* FR=Food responsiveness; EOE=Emotional overeating; EF=Enjoyment of food; SR=Satiety responsiveness; SE=Slowness in eating; EUE=Emotional undereating; FF=Food fussiness; 6=age 6; 8=age 8; 10=age 10; 12=age 12; 14=age 14; *p<.05; **p<.01; ***p<.001
